# How ‘arm-twisting’ by the inducer triggers activation of the MalT transcription factor, a typical signal transduction ATPase with numerous domains (STAND)

**DOI:** 10.1093/nar/gkv158

**Published:** 2015-03-03

**Authors:** Olivier Danot

**Affiliations:** 1 Institut Pasteur, Molecular Genetics Unit, Microbiology Department, F-75015 Paris, France; 2 CNRS, ERL3526, F-75015 Paris, France

## Abstract

Signal transduction ATPases with numerous domains (STAND) get activated through inducer-dependent assembly into multimeric platforms. This switch relies on the conversion of their nucleotide-binding oligomerization domain (NOD) from a closed, ADP-bound form to an open, ATP-bound form. The NOD closed form is stabilized by contacts with the arm, a domain that connects the NOD to the inducer-binding domain called the sensor. How the inducer triggers NOD opening remains unclear. Here, I pinpointed the NOD-arm interface of the MalT STAND transcription factor, and I generated a MalT variant in which this interface can be covalently locked on demand, thereby trapping the NOD in the closed state. By characterizing this locked variant, I found that the inducer is recognized in two steps: it first binds to the sole sensor with low affinity, which then triggers the recruitment of the arm to form a high-affinity arm-sensor inducer-binding site. Strikingly, this high-affinity binding step was incompatible with arm-NOD contacts maintaining the NOD closed. Through this toggling between two mutually exclusive states reminiscent of a single-pole double-throw switch, the arm couples inducer binding to NOD opening, shown here to precede nucleotide exchange. This scenario likely holds for other STANDs like mammalian NLR innate immunity receptors.

## INTRODUCTION

STAND (signal transduction ATPases with numerous domains) are sophisticated ATPases present in the three domains of life, which integrate several signals and build up large multimeric scaffolds upon activation by an inducer (1,2). These multimeric hubs bring together several units of their target molecules, thereby triggering downstream signaling. The pathways in which these proteins intervene are extremely diverse. Well known examples are APAF-1 (proapoptotic factor 1), the mammalian innate immunity NLR proteins and plant disease resistance proteins (2). Another important subfamily of STAND comprises widespread bacterial transcription factors like MalT from *Escherichia coli*, as well as serine-threonine kinases like PknK from *Mycobacterium tuberculosis*.

**Figure 1.**
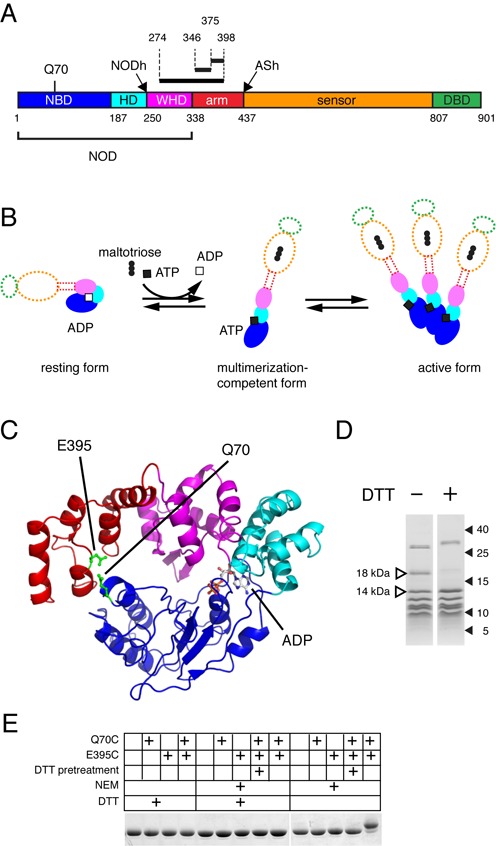
Residue 70 of the MalT NBD can be cross-linked with residues of the arm domain. (**A**) Schematic representation of the MalT primary sequence. NBD, nucleotide binding domain; HD helical domain; WHD, winged-helix domain; DBD, DNA-binding effector domain. In the upper part of the cartoon, the location of residue Q70 and the limits of the peptides (black bars and dotted lines) that were found cross-linked to AET-derivatized C70 after irradiation of HMalT^C-,Q70C^ in the resting form, followed by endoproteinase LysC or trypsin digestion (see text), are indicated. NODh and ASh indicate the proteinase K sensitive hinges (34). (**B**) The STAND activation scheme of MalT (same color coding). The transition from the resting to the multimerization competent form involves three events: inducer binding to the sensor, NOD isomerization, nucleotide exchange. The precise sequence of these events is not known, and the role of the sensor and the arm in this process is unclear (indicated by dotted outlines). (**C**) Model of the MalT structure from aa 1 to 417. Color coding is as in (**A**) except for Q70 and E395, which are represented in green. ADP is colored according to its atoms. (**D**) Analysis of endoproteinase LysC proteolysis products of AET-cross-linked HMalT^C-,Q70C^ (See also Fig. S1B, C, D). Endoproteinase LysC digests of 4 µg HMalT^C-,Q70C^ were analyzed by SDS-PAGE in the presence (+) or absence (-) of DTT. Molecular weight of markers are indicated in kDa, 18 kDa and 14 kDa fragments are highlighted. (**E**) HMalT^Q70C,E395C^ disulfide bond formation detected by SDS-PAGE. Proteins HMalT, HMalT^Q70C^, HMalT^E395C^ and HMalT^Q70C,E395C^ were analyzed by SDS-PAGE in the presence and absence of DTT. The + signs indicate the subsitution(s) harboured by the protein (Q70C, E395C), whether the protein was pretreated with DTT as a first step (DTT pretreatment, see Supplementary Materials and Methods), whether the sample was treated with N-ethylmaleimide (NEM) and whether DTT was present during SDS-PAGE analysis (DTT).

STAND are large, multidomain proteins built according to a conserved scheme (2). Their hallmark is the nucleotide-binding oligomerization domain (NOD) which is basically the nucleotide binding domain-helical domain (NBD-HD) moiety of the AAA+ ATPases supplemented with a winged-helix domain (WHD) at its C-terminus (Figure 1A). A non-conserved sensor domain, which binds the inducer, is connected to the C-terminus of the NOD, either directly or through a helical domain called the arm (or HD2, or SH). The sensor domain is generally composed of repeated units like the leucine-rich (LRR), tetratricopeptide (TPR), WD40 or ankyrin repeat. At either end of the protein, one or several effector domains (e.g. protein recruitment, enzymatic or nucleic acid binding domains) are responsible for downstream signaling.

The status of the NOD, which functions as an ADP/ATP switch, is the main determinant of STAND activity (1,3). In the absence of inducer (the molecule that triggers signaling), the protein is in a monomeric resting state characterized by an ADP-bound closed NOD (Figure 1B), as exemplified by the X-ray structures of APAF1 (4,5) and NLRC4 (6). In this NOD conformation, the WHD closes over the NBD-HD bound ADP, contacting both the ADP molecule and the NBD (5–7 and references therein). In the STAND active form, the NOD is open, ATP-bound and mediates multimerization mainly via head-to-tail association of its NBD-HD moiety, with ATP sandwiched between protomers (8–10). ATP hydrolysis drives the protein back to its resting state and extinguishes the signal if the inducer is no longer present (3,11–13).

How the NOD switch is turned on by the inducer and which role the sensor plays in the process are much less well understood. As shown recently, inducer binding favors a conformation in which the NOD is open (14), but the mechanism underlying this effect is unclear. It has been hypothesized that STAND proteins exist in an autoinhibited state in which the sensor domain prevents activation (15–19) and that inducer binding relieves this autoinhibitory effect (18,20,21). However, recent data suggest that autoinhibition might actually involve both the arm and the sensor: the two crystal structures of resting APAF1 and NLRC4 (4,6) displayed sensor/NBD, but also arm/NBD contacts that need to be disrupted to open the NOD. Moreover, the mechanism whereby inducer binding relieves autoinhibition to allow NOD opening remains elusive. Comparative analysis of the X-ray structure of APAF1 in the resting form and the cryoEM structure of the APAF1 apoptosome identified important reorientations of domains that are required for the transition of the resting form to the inducer-bound, open-NOD form. Yet the sequence of these rearrangements (for instance whether nucleotide exchange occurs before or after NOD opening) and the driving forces that underly these conformational changes are still debated (4,22).

Here, I dissected the early events of signaling by the MalT transcription factor, a typical STAND protein and one of the best characterized members of the family. This protein multimerizes into polydisperse multimers (23,24) in response to the binding of its cognate inducer, maltotriose, to its sensor, which is of the SUPR (superhelical repeats, a TPR variant) type (25,26). Multimerization is the key step allowing the cooperative binding of MalT to the MalT boxes of its target promoters and the recruitment of RNA polymerase, most likely through contacts with the MalT DNA-binding domain (27). To delineate the sequence of events of the signaling pathway leading to NOD opening, the step that commands multimerization, and to dissect the role played by the different domains along that pathway, I trapped the MalT NOD in a closed conformation by covalently locking the arm–NBD interface. By characterizing the properties of the obtained variant, I have shown the existence of a first, low affinity inducer binding step, followed by a second high affinity binding step that is coupled with the opening of the NOD. The arm plays a key role in this process by switching partners from the NBD to the liganded sensor. The body of data available thus far suggest that this arm-based mechanism likely applies to other STANDs, especially the mammalian NLR proteins.

## MATERIALS AND METHODS

### Strains, plasmids, proteins

Plasmid constructs are explained in the Supplementary Material and Methods. All proteins were purified by immobilized metal ion chromatography on Ni^2+^ containing resins, followed by size exclusion chromatography on a Superdex 200 column. Experimental details are given in the Supplementary Material and Methods.

### Cross-linking experiments

HMalT^C-,Q70C^ (20 μM, 2.95 ml) was incubated in buffer B (50 mM Tris–HCl (pH 8.0), 10% sucrose, 0.3 M KCl, 10 mM Mg acetate, 0.1 mM EDTA) + 0.5 mM ATP with 200 μM *S*-[2-(4-azidosalicylamido)ethylthio]-2-thiopyridine (AET) in the dark for 1 h at 24°C. The protein was rid of unreacted AET by filtering through a HiTrap Desalting column equilibrated in buffer B + 0.5 mM ATP. The eluate was diluted to 5 μM in 1 ml samples in a 24-well tissue-culture test plate, and irradiated with a B100AP UV lamp (UVP, Upland, USA) for 3 min to allow photocross-linking. The cross-linked HMalT^C-,Q70C^-AET protein was concentrated to 750 μl, 1 mM maltotriose was added and 360 μl were passed through a Superdex 200 10/300 GL column (GE Healthcare) equilibrated in buffer B + 0.5 mM ATP + 1 mM maltotriose. The protein eluted as two partially overlapping peaks corresponding to multimeric and monomeric species. Fractions corresponding to the monomeric species were pooled and subjected to endoproteinase LysC proteolysis followed by N-terminal microsequencing or trypsinolysis, HPLC and SELDI-TOF (see Supplementary Material and Methods).

### Half-life measurements

Twenty microliters protein (HMalT^Q70C,E395C^ (107 μM) or HMalT (101 μM)) were preincubated for 15 min at 20°C with 2 mM dithiothreitol to reduce disulfide bonds and run through a Superdex 200 PC 3.2/30 column (GE Healthcare) equilibrated in buffer C (20 mM HEPES (pH 8.0), 10% sucrose, 0.2 M KI, 10 mM Mg acetate, 0.1 mM EDTA) + 0.1 mM DTT to ensure that the HMalT^Q70C,E395C^ disulfide bond is reduced and to get rid of free ATP. Fractions eluting between 1.35 and 1.5 ml, corresponding to the monomeric form of the protein, were collected and pooled. Protein from the pool was immediately incubated at 8.6 to 10.1 μM with radiolabeled ADP (10 μM [α-^32^P] ADP, ∼2 μCi/ml) for 2 h at 25°C in buffer C containing 0.1 mM DTT, 1.3 mM Tris–HCl pH 8.0, 7 mM NaCl, 200 μg/ml acetylated bovine serum albumin (BSA), in order to load it with radiolabeled ADP. The mix was divided in two samples (+ and –) which were buffer exchanged by two passages through 0.7 ml Protein Desalting Columns (Thermo Scientific) and immediate 1.5 dilution. The final buffer was buffer B containing 10 μM [α-^32^P] ADP (∼2 μCi/ml) and 400 μg/mL BSA, with (+ mix) or without (– mix) 1 mM DTT. Proteins were incubated in this buffer for 1 h at 25°C, allowing possible disulfide bonds to reform only in the –DTT mix. Two 10 μl samples were subjected to the filter-binding assay described below, immediately before cold ADP (1 mM final concentration) and maltotriose when required (20 mM final concentration) were added, with (+ mix) or without (– mix) DTT (1 mM final concentration). At various times after cold ADP addition, 10 μl samples were filtered through a disk of polyvinylidene difluoride membrane (Immobilon P, pore size 0.45 μm, diameter 26 mm prepared as specified by the manufacturer (Millipore)) mounted on a vacuum filtration mannifold. The filter was immediately washed with 2 ml ice-cold buffer C. Filters were counted in 10 ml of BCS scintillation cocktail (GE Healthcare).

### ADP binding assay

Twenty microliters HMalT^Q70C,E395C^ (107 μM) or HMalT (77 μM) were rid of free ATP by filtration through a Superdex 200 PC 3.2/30 column (GE Healthcare) equilibrated in buffer C. Fractions eluting between 1.35 and 1.5 ml, corresponding to the monomeric form of the protein, were collected. The obtained protein was immediately incubated at 25°C and at a concentration of 5.3–6.2 μM with 10 μM [α-^32^P] ADP (∼2 μCi/ml) in buffer C containing 1.7 mM Tris–HCl pH 8.0, 8 mM NaCl, 200 μg/ml BSA. Ten microliters volumes were processed at different times exactly as described above.

### Analytical exclusion chromatography

Proteins were preincubated for 15 min at 20°C and injected on a superdex 200 PC 3.2/30 (GE Healthcare) mounted on an EttanLC system (GE Healthcare) and run at 20°C. Unless noted otherwise, preincubation and equilibration buffers were: 55 mM Tris–HCl (pH 8.0), 1.7% sucrose, 33 mM KI, 24 mM tripotassium citrate, 9 mM Mg acetate, 0.09 mM EDTA, 0.14 mM ATP, 10 μM pyridoxal 5′-phosphate and 50 mM Tris–HCl (pH 8.0) containing 33 mM tripotassium citrate, 10 mM Mg acetate, 0.1 mM EDTA, 0.1 mM ATP, 10 μM pyridoxal 5′-phosphate, respectively. Buffers were supplemented with maltotriose and DTT when indicated. The column was calibrated with three standard globular proteins: thyroglobuline (669 kDa), β-amylase (200 kDa) and BSA (66 kDa).

### Limited proteolysis

Proteins (4 μg) were preincubated for 10 min at 25°C in a buffer containing 50 mM HEPES (pH 8.0), 5 mM Tris–HCl (pH 8.0), 1% sucrose, 20 mM KI, 0.27 mM KCl, 10.1 mM Mg acetate, 0.44 mM ATP, 0.01 mM EDTA. When indicated, maltotriose was present at 20 mM and DTT at 2 mM. Proteinase K was added, and the reaction was allowed to proceed for 30 min at 25°C. The reaction was stopped by diluting with 3 volumes of TCA 22%. The precipitate was recovered by centrifugation, washed with cold acetone, dried, redissolved in sample buffer containing 70 mM DTT and analyzed by SDS-PAGE.

### Measurement of the affinity of HMalT^Q70C,E395C^ for maltotriose

Limited proteolysis of HMalT or HMalT^Q70C,E395C^ was performed as above with the following differences: proteins were digested at a single proteinase K/HMalT ratio (1:267), in the presence of increasing maltotriose concentrations, and only 0.6 μg of each sample was analyzed by SDS-PAGE. The gels were stained with Oriole™ fluorescent gel stain (Bio-Rad). The progressive disappearance of the 45–50 kDa bands characteristic of the absence of maltotriose was quantified using the ImageJ software (28). The protein fractional saturation (calculated by assuming that full saturation is achieved at 50 mM maltotriose) was plotted against maltotriose concentration and fitted to a binding isotherm by non linear least-squares fitting.

### Fluorescence spectroscopy

Fluorescence measurements were performed on a PTI (Lawrenceville, NJ, USA) QuantaMaster400 spectrofluorometer at 20°C on 1.1 ml samples containing 1 μM HMalT^336–806^ in a 20 mM Tris–HCl (pH 8.0), 0.2 M KCl degassed buffer. The excitation wavelength was 295 nm, excitation and emission bandwidths were 0.8 and 5 nm, respectively. Maltotriose and maltose binding studies were carried out by allowing the protein to equilibrate at different ligand concentrations for 15 min and recording fluorescence emission at 323 nm during 120 s. Each data point is the average of these 120 measurements, and error bars indicate the corresponding standard deviation. The *K*_D_ was calculated by fitting the relative fluorescence increase to the quadratic binding equation:

\begin{eqnarray*} &&({F} - {F}_0 )/{F}_0 =\nonumber \\ &&{C}({K}_{D} + {P}_0 + {M}_0 - (({K}_{D} + {P}_0 + {M}_0 )^2 - 4{P}_0 {M}_0 )^{1/2} ) \end{eqnarray*}

where *F*_0_ is the fluorescence in the absence of maltotriose, *C* a proportionality constant, *P*_0_ is the total protein and *M*_0_ is the total maltotriose concentration.

## RESULTS

### Construction of a derivatized monocysteine variant of MalT to probe NBD-arm contacts

Contacts between the arm and the NBD have been observed in STAND protein structures exhibiting a closed NOD (4–6) and were suggested to be involved in the stabilization of the closed form of the NOD (6). To probe for such contacts in the MalT protein, I generated a model of the MalT NOD-arm in the resting form (Figure 1C) with Modeller (29), using an APAF1/MalT alignment obtained with the HHpred suite (30).

To test the validity of the NBD-arm interface predicted by the model, a site-specific probe approach was taken: I grafted a cross-linking agent at a position on the surface of the NBD facing the arm, activated it to allow cross-linking with spatially close atoms, and identified the cross-linked targets. Namely, a His-tagged variant of the MalT protein (HMalT^C-,Q70C^) with a unique cysteine at position 70, which lies in the NBD and faces the arm in the model (Figure 1C), was produced. The resulting protein behaved like the wt: it underwent maltotriose-dependent multimerization in the presence of ATP, although to a lesser extent than His-tagged MalT (HMalT) (Supplementary Figure S1A). A bifunctional, photoactivatable, thiol-reactive, cleavable cross-linking agent, *S*-[2-(4-azidosalicylamido)ethylthio]-2-thiopyridine (31), was reacted with resting HMalT^C-,Q70C^ to derivatize its unique cysteine. Photoactivation of the aryl-azide moiety of AET is expected to generate a nitrene group that either reacts with primary amines or inserts into C–H and N–H sites, yielding a 15 Å cross-link (cysteine Cα/cross-linked atom distance). The derivatized protein was irradiated in its resting form to trigger photocross-linking. It was then submitted to size-exclusion chromatography in the presence of ATP and maltotriose, i.e. in multimerization conditions. The monomeric species, which were liable to contain forms with a cross-linked NOD module unable to open, were subjected to endoproteinase LysC digestion in mild denaturing conditions, followed by SDS-PAGE in the presence or absence of dithiothreitol (DTT), a reducing agent able to cleave AET. One band with a predicted MW of 18 kDa was present only in the absence of DTT (Figure 1D). Microsequencing suggested that it contained peptide 46–99 of HMalT^C-,Q70C^ cross-linked to peptide 274–398 (Figure 1A, see also Supplementary Material and Methods). The locations of the cross-links were narrowed down by trypsinolysis of the fragments contained in the 18 kDa band, followed by HPLC. Two types of cross-links were identified by surface enhanced laser desorption ionization-time of flight mass spectrometry (SELDI-TOF) and microsequencing of the HPLC purified tryptic peptides: they bridged the C70-containing tryptic fragment 57–73 with either peptide 346–374 or peptide 375–398 of the arm (Figure 1A, Supplementary Figure S1B, C and D). This indicates that in the resting form of the wt protein, Q70 lies in physical proximity to the arm, as predicted by the model.

### A disulfide bond forms spontaneously between two cysteines introduced at positions 70 and 395 of MalT

To be able to freeze the MalT protein quantitatively in a closed NOD conformation, I introduced a cysteine at positions Q70 and E395 of HMalT, whose α-carbons lie 9 Å apart in the model, generating HMalT^Q70C,E395C^. The variants with one cysteine substitution (HMalT^Q70C^, HMalT^E395C^) were also produced.

To ascertain that HMalT^Q70C,E395C^ contained a disulfide bond, proteins HMalT, HMalT^Q70C^, HMalT^E395C^ and HMalT^Q70C,E395C^ were purified in the absence of a reducing agent, denatured, treated with N-ethyl maleimide (NEM) to block free cysteines and subjected to SDS-PAGE with or without a reducing agent (DTT). HMalT, HMalT^Q70C^, HMalT^E395C^ migrated at the same level irrespective of the presence of DTT during electrophoresis (Figure 1E). However, HMalT^Q70C,E395C^, whose reduced form also migrated as the wt, displayed a major band migrating more slowly in non-reducing conditions. This pattern was abolished when the protein was reduced before denaturation. In conclusion, purified HMalT^Q70C,E395C^ is spontaneously and reversibly oxidized in nearly stoichiometric amounts and this oxidation requires both C70 and C395. This indicates that HMalT^Q70C,E395C^ harbors a C70-C395 disulfide bridge when purified in non-reducing conditions.

### The C70-C395 disulfide bond locks the NOD in the closed state

To characterize the oxidized HMalT^Q70C,E395C^ variant, I first checked whether its NOD was in the closed state irrespective of the presence of inducer, as expected from the locations of Q70 and E395 in the model. To that purpose, I measured the half-life of the HMalT^Q70C,E395C^-ADP complex. Indeed, the release of ADP from the HMalT–ADP complex is extremely dependent on the conformation of the NOD: ADP-complexes of the resting form and open NOD forms (measured in reducing conditions) are characterized by half-lifes of ∼3 h and ∼2 min, respectively (14).

**Figure 2.**
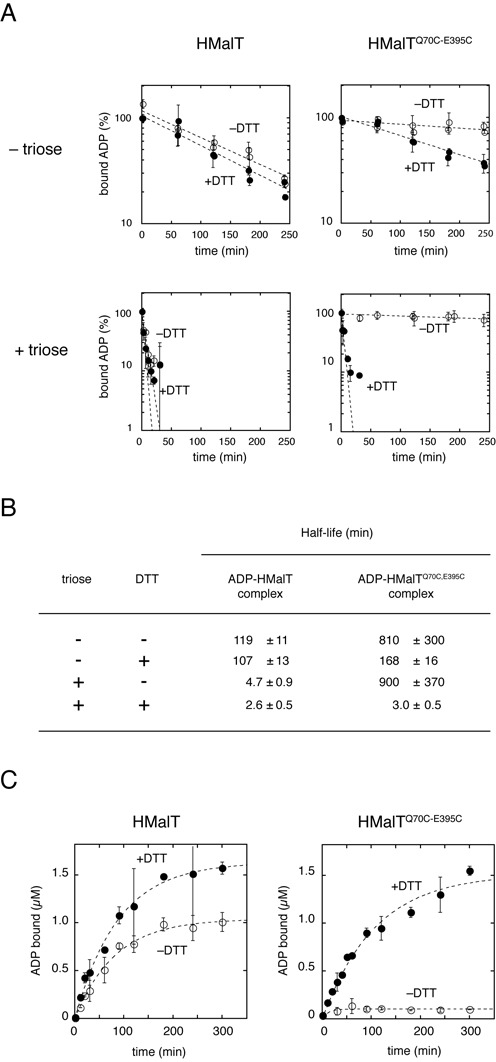
The disulfide bond of oxidized HMalT^Q70C,E395C^ traps the protein in a long-lived complex with ADP. (**A**) ADP is sequestered by oxidized HMalT^Q70C,E395C^ irrespective of the presence of the inducer. Dissociation of the complexes formed by [α-^32^P] ADP and HMalT or HMalT^Q70C,E395C^ was monitored in the presence and absence of maltotriose by a filter-binding assay, under reducing and non-reducing conditions. Data points and error bars represent the average and standard deviation of measurements taken at the same time point in three (HMalT^Q70C,E395^) or two (HMalT) independent experiments. Dotted lines correspond to the best fit of a pseudo first-order equation to the data. Note that the + and − triose y-axis scales differ. (**B**) Half-lives of the complexes in the different conditions assayed. Half-lives ± standard errors were derived from the (A) plots by non-linear least-squares fitting (see above). (**C**) Residual ADP-binding in the absence of inducer is abolished by the disulfide bond of oxidized HMalT^Q70C,E395C^. [α-^32^P] ADP binding to HMalT (6.2 μM) or HMalT^Q70C,E395C^ (5.2 μM) in the presence or absence of DTT was measured by a filter binding assay. Data points and error bars represent the average and standard deviation of measurements taken at the same time point in two independent experiments.

After HMalT and HMalT^Q70C,E395C^ loading with radiolabeled ADP, the complexes were challenged by cold ADP in excess, in reducing or non-reducing conditions, with or without inducer, and subjected to a filter-binding assay. The HMalT protein behaved in non-reducing conditions as in reducing conditions: its complexes with ADP were long-lived in the absence of inducer, reflecting a resting conformation, and short-lived in the presence of inducer, characteristic of an open NOD (Figure 2A and B). The reduced HMalT^Q70C,E395C^ behaved similarly, which indicates that the Q70C and E395C substitutions by themselves do not affect the ability of the NOD to retain ADP in the presence and absence of inducer. Strikingly however, the oxidized HMalT^Q70C,E395C^ formed very stable complexes with ADP, irrespective of the presence of maltotriose; their *t*_1/2_ (>500 min) are longer than that of the resting HMalT–ADP complex (*t*_1/2_ = 119 ± 11 min). Altogether, these results establish that the NOD of oxidized, inducer-bound HMalT^Q70C,E395C^ is closed, similarly to the wild-type in its resting form.

Moreover, the dramatic stabilization of the MalT–ADP complex that characterized the disulfide bonded variant suggests that residual ADP release from resting wt MalT results from transient NOD opening caused by thermal fluctuations. To confirm this interpretation, I examined the binding kinetics of radiolabeled ADP to the proteins in the absence of inducer. Slow ADP binding was indeed observed for reduced HMalT, for oxidized HMalT (with a marginal difference probably due to partial inactivation by the formation of incorrect disulfide bonds), and for reduced HMalT^Q70C,E395C^ (Figure 2C). By contrast, virtually no ADP binding occurred with oxidized HMalT^Q70C,E395C^, indicating that a covalently locked closed NOD is a virtually impassable barrier for ADP.

### The C70–E395 disulfide bond prevents the adoption of the active form

**Figure 3.**
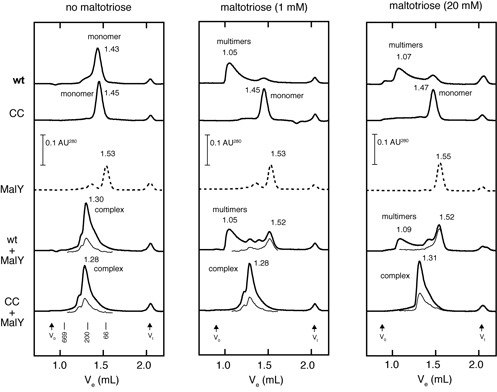
The HMalT^Q70C,E395C^ C70-C395 disulfide bond prevents inducer-dependent multimerization and inducer-triggered dissociation of the complex of HMalT^Q70C,E395C^-MalY (MalY is a protein inhibitor of MalT which specifically binds MalT in its resting form). Purified proteins (10 μM in monomers) were incubated for 15 min at 20°C and filtered through a Superdex 200 column in the absence of a reducing agent. CC stands for HMalT^Q70C,E395C^. Maltotriose was present both in the incubation and in the running buffer at the indicated concentration. Absorbance at 390 nm (multiplied by 15, represented by hairline traces in the 1.08–1.6 ml range) is indicated for cofiltration experiments to monitor the presence of MalY (11). Activated MalT generates polydisperse head-to-tail multimers in fast equilibrium with each other, hence the typical trailing shape of the HMalT curves in the presence of maltotriose (center and right panel, top).

Trapping NOD in the closed state is expected to interfere with MalT activation by the inducer. This prediction was tested by monitoring inducer-dependent multimerization of oxidized HMalT^Q70C,E395C^ by size exclusion chromatography in the presence of ATP (free ATP does not alter the monomeric state of the ADP-bound resting form in the absence of inducer (24)). Multimerization of HMalT (Figure 3, left and center panels, top curves) in the absence of DTT was similar to that typically observed for MalT in reducing conditions ((24), see also Supplementary Figure S2). By contrast, HMalT^Q70C,E395C^ did not undergo inducer-dependent multimerization in non-reducing conditions. The absence of multimerization was clearly due to the presence of the disulfide bridge, as shown by the fact that reduced HMalT^Q70C,E395C^, oxidized HMalT^Q70C^ and oxidized HMalT^E395C^ multimerized in response to the inducer as the wt (Supplementary Figures S2 and S3). The absence of response of disulfide-bridged HMalT^Q70C,E395C^ to the inducer could be explained by a low affinity for maltotriose (see below), therefore the protein was subjected to size exclusion chromatography at a higher inducer concentration (Figure 3, right panel). HMalT^Q70C,E395C^ remained monomeric, which shows that locking NOD in the closed form completely prevents inducer-dependent multimerization.

MalY is a well characterized protein inhibitor of MalT which specifically binds MalT in its resting form by interacting with the NOD region (11,12,32,33). Maltotriose is known to compete MalY away in the presence of ATP. To determine the step at which the activation pathway was blocked, I asked whether MalY formed a complex with oxidized HMalT^Q70C,E395C^ and whether maltotriose could dissociate that complex. Size exclusion chromatography of a stoichiometric mixture of MalY and HMalT^Q70C,E395C^ in the presence of ATP and in the absence of a reducing agent gave one peak corresponding to the MalY–HMalT^Q70C,E395C^ complex, and this complex could not be dissociated by maltotriose, whether at 1 or 20 mM (Figure 3 and Supplementary Figure S4). By contrast, the HMalT–MalY complex formed in the same conditions was readily dissociated by addition of 1 mM maltotriose (Figure 3). Controls run in the presence of DTT again showed that resistance of the HMalT^Q70C,E395C^–MalY complex to dissociation by the inducer was due to the presence of the disulfide bond (Supplementary Figure S2).

Altogether, these results show that in the presence of inducer, oxidized HMalT^Q70C,E395C^ has all the characteristics of the resting form: it is monomeric and interacts with MalY. Therefore, oxidized HMalT^Q70C,E395C^ either remains in the resting state irrespective of the presence of the inducer, or adopts a slightly different conformation, wherein the NOD is still closed, in response to inducer binding.

### The C70-E395 disulfide bond traps the protein at an intermediate stage of the activation pathway in the presence of inducer

**Figure 4.**
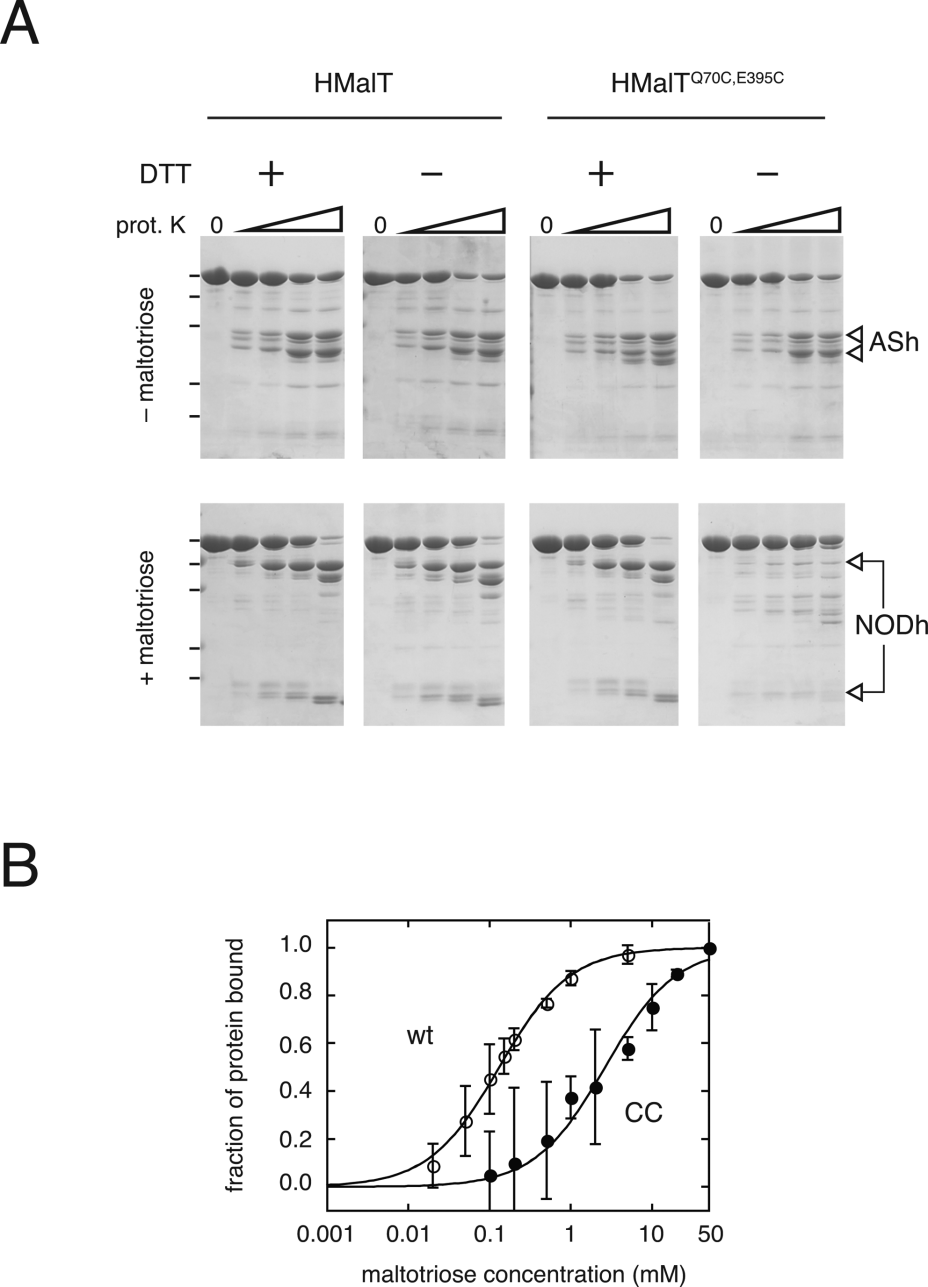
Characterization of the preactivated intermediate. (**A**) Inducer-bound non-reduced HMalT^Q70C,E395C^ adopts an intermediate conformation with protease-resistant NOD and AS hinges. Limited proteolysis of HMalT and HMalT^Q70C,E395C^ by proteinase K with or without inducer, under reducing or non-reducing conditions. For each set of conditions, increasing proteinase K/HMalT w/w ratios were used: 0, 1:1067, 1:533, 1:267, 1:133. The position of protein markers of 100, 70, 55, 35 and 25 kDa are indicated by black dashes on the left of the figure. Location of proteolysis products corresponding to AS and NOD hinge cleavage are indicated by open arrows on the right (ASh and NODh, respectively). (**B**) Non-reduced HMalT^Q70C,E395C^ binds maltotriose with low affinity. Maltotriose saturation curves for HMalT (wt, open circles) and HMalT^Q70C,E395C^ (CC, solid circles) obtained from quantification of the bands corresponding to AS hinge proteolysis by proteinase K in the absence of reducing agent. Data points and error bars represent the average and standard deviation of three independent experiments. *K*_D_ of 2.6 ± 0.5 mM for HMalT^Q70C,E395C^ and 131 ± 12 μM for HMalT were extracted from these data by non linear least-squares fitting (solid lines).

To distinguish between these two hypotheses, inducer binding was assayed using sensitivity to proteolysis as a probe of protein conformation. The conversion of the resting form to the multimerization competent form affects proteinase K sensitivity of two hinge regions in the wild-type protein (34): the hinge connecting the arm and the sensor (ASh, see Figure 1A), and the NOD hinge, between the HD and the WHD (NODh). In the resting form of wt MalT, proteinase K cleaves ASh but not NODh, which generates two fragments of 50 and 45 kDa (apparent MW). Conversely, in the multimerization-competent form, proteinase K cleaves NODh but not ASh, yielding 70 and 25 kDa fragments. The status of the NOD hinge is therefore a probe of the conformation of the NOD (proteolysis resistant/closed, proteolysis sensitive/open) while the AS hinge state reflects the status of the sensor (proteolysis sensitive/unliganded, proteolysis resistant/inducer-bound). As expected, the reduced and oxidized forms of HMalT (Figure 4A), HMalT^Q70C^, HMalT^E395C^ (Supplementary Figure S5) and the reduced HMalT^Q70C,E395C^ (Figure 4A) behaved similarly, giving proteolytic profiles in the absence and the presence of inducer that are characteristic of the resting and active form, respectively. In the absence of inducer, the oxidized HMalT^Q70C,E395C^ variant displayed the resting form proteolytic profile. By contrast, in the presence of 20 mM maltotriose, both the AS and NOD hinges of oxidized HMalT^Q70C,E395C^ were proteolysis resistant (Figure 4A). Proteolysis resistance of the NOD hinge was expected since the NOD is closed, as demonstrated above. Most interestingly however, proteolysis resistance of the AS hinge in the presence of maltotriose indicates that the sensor of oxidized HMalT^Q70C,E395C^ binds the inducer. Inducer binding and NOD opening are therefore uncoupled by the presence of a disulfide bond between C70 and C395. Inducer-bound oxidized HMalT^Q70C,E395C^ typifies an intermediate in the pathway of activation that is characterized by a closed NOD and a liganded sensor and that I hereafter call the preactivated state.

### The preactivated state arises through a low affinity inducer binding step

Affinity of maltotriose for the isolated MalT sensor is lower than for the whole protein (34), suggesting that the sensor alone does not contain all the determinants of maltotriose binding. One possiblity is that the maltotriose binding-site does not preexist in the resting form but is assembled from the sensor and other parts of the protein at a given step in the STAND activation pathway. I therefore asked whether the complete maltotriose-binding site is already present in the preactivated intermediate, by quantifying the disappearance of the 45–50 kDa limited proteolysis MalT fragments as a function of maltotriose concentration (Figure 4B). The apparent dissociation constant for maltotriose binding obtained by this procedure was 2.6 ± 0.5 mM for the oxidized HMalT^Q70C,E395C^, in contrast to a value of 131 ± 12 μM obtained for the wt. This 20-fold difference suggests that locking the NOD-arm region in the closed conformation prevents the protein from binding maltotriose with high affinity, while allowing low affinity inducer binding to the sensor. Since the value obtained for maltotriose binding to disulfide-bonded HMalT^Q70C,E395C^ is close to that measured for the isolated sensor domain (34), an attractive hypothesis is that in the wild-type protein, high-affinity inducer binding involves cooperation of the sensor with another domain, and that this cooperation is abolished when the arm is bridged to the NBD by a disulfide bond. The arm is an obvious candidate to be the helper domain.

### The arm-sensor polypeptide binds maltotriose with high affinity

**Figure 5.**
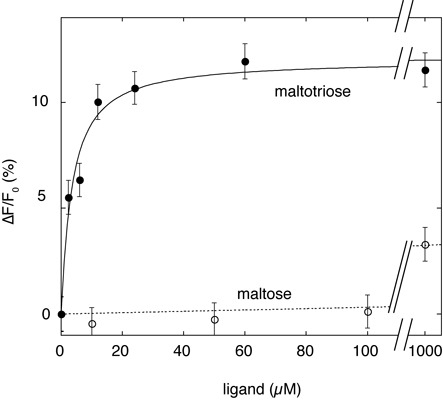
Affinity of the arm-sensor polypeptide HMalT^336–806^ for maltotriose. Representative maltotriose (filled circles) or maltose (open circles) binding curves of HMalT^336–806^ (1 μM) obtained by monitoring the fluorescence emission change at 323 nm, after excitation at 295 nm, are presented. The fitting curve used to extract the dissociation constant for maltotriose binding is shown (continuous line). The *K*_D_ indicated represents the average of three independent experiments.

To test this hypothesis, the affinity of the purified arm-sensor polypeptide (HMalT^336–806^) for maltotriose was studied by fluorescence spectroscopy. As already shown for the isolated sensor domain, intrinsic tryptophane fluorescence of HMalT^336–806^ was enhanced by maltotriose, and the fluorescence emission maximum was shifted toward shorter wavelengths (Supplementary Figure S6). Maltotriose binding to HMalT^336–806^ was monitored by recording the fluorescence emission of the polypeptide in the presence of increasing maltotriose concentrations at the excitation wavelength that gave the maximal fluorescence change (Figure 5). Data were fitted to a quadratic equation, giving a *K*_D_ value of 2.7 ± 1.2 μM, which was 1000 times less than that measured for the isolated sensor (34) or for disulfide-bonded HMalT^Q70C,E395C^ protein. The interaction was highly specific since 100 μM maltose did not alter the intrinsic fluorescence (Figure 5). Therefore, the arm is a key component of the high-affinity inducer binding site. The inability of the arm to cooperate with the sensor for inducer binding when the NOD is locked in the closed conformation suggests that it functions as a switch toggling between two states: one in which it participates to inducer binding, the other in which it helps maintaining the NOD closed.

## DISCUSSION

### The structure of the arm domain

Here, a model of the NOD-arm region of MalT in the closed form was obtained using an APAF1 NOD-arm X-ray structure as a template (Figure 1C) and was validated by chemical cross-linking data. Indeed, in the model, atoms within 15 Å (the expected cysteine Cα/cross-linked atom distance for AET cross-links, as deduced from (31)) of Q70 Cα lie either in the NBD, or in one of three segments of the arm, 357–365, 382–397 and 409–413, with one exception, residue 278 of the WHD. AET cross-linking of cysteine 70 of HMalT^C-,Q70C^ with regions 346–374 and 375–398 of the arm is in good agreement with these observations. The model is also substantiated by the fact that cysteine residues introduced at position 70 and 395 spontaneously build a disulfide bridge within the protein in the resting form, as judged from its long-lived ADP retention and its interaction with the MalY inhibitor. There again, the Cα–Cα distance between residues 70 and 395 in the model is 9 Å, while statistical data on cysteines engaged in a disulfide bond indicate a Cα–Cα distance range of 4–9 Å (35). Altogether, these results suggest that the structure of the arm and its location with respect to the other subdomains of the NOD in the resting form of MalT are faithfully described by the model presented here (Figure 1C).

### The arm-based partner switch mechanism

The major finding of this work is that in MalT, the arm plays a dual role in the control of the STAND switch: it is involved in both the stabilization of the resting form and in inducer binding.

Indeed, the spatial proximity between Q70 of the NBD and regions 346–374 and 375–398 of the arm demonstrated here sheds a new light on the fact that many mutations leading to a constitutive transcription activation by MalT fall either in the vicinity of Q70 (14) or within these regions of the arm (32,36,37). Altogether, these results strongly suggest that residues of these two regions physically interact to maintain NOD closed in the resting form, as observed for APAF1 (5) and NLRC4 (6).

**Figure 6.**
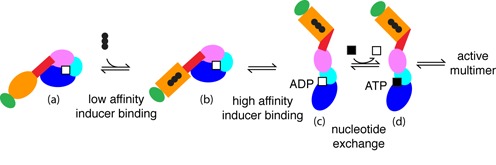
Transition from the resting form (**a**) to the multimerization competent form (**d**) in the MalT STAND protein. The two inducer binding steps and the preactivated intermediate (**b**) are presented. High affinity inducer binding involves both the sensor and the arm, and is incompatible with arm-NBD contacts, so that unlocking of the NOD ensues (**c**). Note that this incompatibility can have two origins: either a competition between the liganded sensor and the NBD for the same site on the arm (as illustrated here), or the toggling of the arm between two conformations favoring interactions either with the NBD or with the liganded sensor. In the end, only when NOD is open can nucleotide exchange occur, leading to the multimerization competent form.

Second, the 1000-fold difference in affinity for maltotriose between the isolated MalT sensor and the arm-sensor polypeptide (2.5 mM versus 2.7 μM) demonstrates that the arm participates in the binding of the inducer, either by interacting directly with the latter or by establishing favorable interactions with the liganded sensor. The arm interacts therefore with two crucial elements of the STAND commutator, the NBD in the resting form and the liganded sensor in the active form. Moreover, this work also shows that these interactions exclude each other: the arm of a protein with a NOD locked in the closed form (oxidized HMalT^Q70C,E395C^) is unable to assist the sensor for inducer binding. The arm is most likely in equilibrium between two states, one favoring interactions with the NBD, the other favoring interactions with the liganded sensor (Figure 6). Activation of the NOD switch by the inducer occurs through harnessing the free energy of arm/liganded sensor interactions to disrupt arm-NBD interactions, which allows spontaneous NOD opening. This hypothesis is supported by the intermediate affinity of the wt protein for maltotriose (in the 100 μM range): in the wt MalT context, the arm toggles between the two states, so that the resulting affinity for the inducer is a weighted average of the low affinity that characterizes the protein with a locked closed NOD and the high affinity of the multimer with open NODs.

Most appealingly, a similar partner-switch mechanism involving an arm-sensor bipartite inducer binding site might be at play in other arm-containing STAND proteins, such as mammalian NLR proteins. On one hand, the arm clearly appears to be involved in NOD closure in these proteins: the NLRC4 structure shows arm-NBD interactions whose disruption results in constitutivity (6), numerous NOD1 and NOD2 arm mutations also lead to constitutivity, while arm mutations of NLRP3 were associated with auto-inflammatory diseases (18). On the other hand, conflicting results were obtained regarding inducer-binding to the LRR sensor of mammalian NLR proteins. The LRR was shown to bind the inducer in the case of NLRX1 (38), and to harbor determinants of the ligand binding site in the case of NOD1 and NOD2 (18) while in cocrystallization or soaking experiments involving the NLRP1 sensor and its cognate inducer muramyl dipeptide, no electron density corresponding to the inducer was detected in the crystals (39). The answer to that conundrum might be that, as shown for MalT, the inducer-binding site of NLR proteins is bipartite and harbored by the sensor and the arm, so that affinity of the inducer for one isolated half-site might be too low to be detected. Interestingly, domain swap experiments on murine NAIP proteins (40) do reveal that specificity determinants involved in inducer binding are harbored by a region spanning the HD, WHD, arm and the beginning of the sensor (the end of the specificity region, which was considered to be a linker between the arm and the sensor, contains sequences identified as LRR motifs by LRRsearch (41) and therefore likely represents the N-terminal part of the sensor). All these observations suggest that the arm partner switch discovered in MalT might be a widespread feature of arm-containing STAND proteins.

Noteworthy, in STAND proteins devoid of the arm, a subdomain of the WHD might play the same role, as suggested by the observation that two neighboring residues of the WHD of R proteins Rx1 and Gpa2 play different roles in the STAND activation process, one being involved in autoinhibition and the other in inducer-triggered activation (42).

### Inducer binding, a two step process

It was already known that inducer binding was the first step of the MalT activation process and caused the opening of NOD (14). However, the possibility of a concerted mechanism in which inducer binding and NOD opening occurred simultaneously was not excluded. Another point of debate about STAND proteins was whether ADP release preceded NOD opening or vice versa (4,22). Here, with the isolation of the preactivated intermediate by disulfide trapping and its characterization, I have delineated the sequence of the major steps of the activation pathway up to the multimerization-competent form (Figure 6). In a first step, maltotriose binds to the sensor of the protein in the resting form with low affinity, yielding the preactivated intermediate. A conformational change then couples the assembly of a high-affinity arm-sensor inducer-binding site with the disruption of NBD–arm contacts that stabilized the closed NOD, as discussed above. In the absence of arm–NBD contacts, the NOD spontaneously opens, a step absolutely required for nucleotide exchange. An interesting consequence of the latter observation is that freezing NOD in the closed state is sufficient to silence the signaling pathway governed by any given STAND protein. Molecules that bind to both the NBD and the arm of a STAND with high affinity are expected to be strong inhibitors of the cognate pathway. In that respect, it is worth noting that most negative effectors of STAND proteins so far characterized act by stabilizing the resting form (33,43–45).

Here, I have provided strong evidence that inducer-binding is a two-step process. Such a low-affinity initial inducer binding followed by a tightening of the interaction may also be a general feature of STAND proteins. For instance, there are hints of a two-step binding of cytochrome *c* by APAF1. The two WD40 lobes of the APAF1 sensor were splayed in the structure of the resting form (4), but closed over a cytochrome c molecule in a cryo-electron microscopy-derived model of the active apoptosome (46). Furthermore, the conformation of the sensor lobes bound to cytochrome *c* is sterically incompatible with the resting form because of a clash between the NBD and one of the WD40 propellers (4,46). Hence, cytochrome *c* probably binds suboptimally to the sensor with open lobes (e.g. to one of the two lobes, which might be analogous to sensor binding by maltotriose in MalT) in a first step, before the two lobes come together to create a higher affinity site. The latter step is only possible after opening of the NOD, suggesting a coupling between high affinity inducer binding and NOD opening, similar to what is observed with MalT.

Many STAND proteins govern pathways leading to irreversible consequences on the cell fate, like inflammation and apoptosis in metazoans. In prokaryotes, at least some STANDs are at the heart of regulatory networks involved in coping with hostile (host infection) or highly competitive (intestine colonization) environments, and are therefore expected to require precise triggering. For instance, the *M. tuberculosis* PknK serine–threonine kinase is involved in growth control and survival during early infection (47,48). MalT itself regulates genes that are important for intestinal colonization by *E. coli* (49), probably through their role in maltose catabolism (50) and glycogen pool management; it is also involved in the control of virulence factor synthesis in *Vibrio cholerae* (51) or of a global stringent-like response to bronchoalveolar fluid in *Actinobacillus pleuropneumoniae* (52).

In these systems, wrong decisions are presumably highly detrimental to the cell or to the multicellular organism to which it belongs. The activation mechanism described here for MalT, in which the transition of a low-affinity to a high-affinity inducer binding site leads to the formation of the active form, is expected to give a fast and specific response to the inducer. Formation of the low-affinity complex can be viewed as a proof-reading step preventing improper signaling.

## SUPPLEMENTARY DATA

Supplementary Data are available at NAR Online.

SUPPLEMENTARY DATA

## References

[1] Danot O., Marquenet E., Vidal-Ingigliardi D., Richet E. (2009). Wheel of life, wheel of death: a mechanistic insight into signaling by STAND proteins. Structure.

[2] Leipe D.D., Koonin E.V., Aravind L. (2004). STAND, a class of P-Loop NTPases including animal and plant regulators of programmed cell death: multiple, complex domain architectures, unusual phyletic patterns, and evolution by horizontal gene transfer. J. Mol. Biol..

[3] Tameling W.I., Vossen J.H., Albrecht M., Lengauer T., Berden J.A., Haring M.A., Cornelissen B.J., Takken F.L. (2006). Mutations in the NB-ARC domain of I-2 that impair ATP hydrolysis cause autoactivation. Plant Physiol..

[4] Reubold T.F., Wohlgemuth S., Eschenburg S. (2011). Crystal structure of full-length Apaf-1: how the death signal is relayed in the mitochondrial pathway of apoptosis. Structure.

[5] Riedl S.J., Li W., Chao Y., Schwarzenbacher R., Shi Y. (2005). Structure of the apoptotic protease-activating factor 1 bound to ADP. Nature.

[6] Hu Z., Yan C., Liu P., Huang Z., Ma R., Zhang C., Wang R., Zhang Y., Martinon F., Miao D. (2013). Crystal structure of NLRC4 reveals its autoinhibition mechanism. Science.

[7] van Ooijen G., Mayr G., Kasiem M.M., Albrecht M., Cornelissen B.J., Takken F.L. (2008). Structure-function analysis of the NB-ARC domain of plant disease resistance proteins. J. Exp. Bot..

[8] Qi S., Pang Y., Hu Q., Liu Q., Li H., Zhou Y., He T., Liang Q., Liu Y., Yuan X. (2010). Crystal structure of the *Caenorhabditis elegans* apoptosome reveals an octameric assembly of CED-4. Cell.

[9] Yuan S., Yu X., Asara J.M., Heuser J.E., Ludtke S.J., Akey C.W. (2011). The holo-apoptosome: activation of procaspase-9 and interactions with caspase-3. Structure.

[10] Yuan S., Yu X., Topf M., Dorstyn L., Kumar S., Ludtke S.J., Akey C.W. (2011). Structure of the Drosophila apoptosome at 6.9 Å resolution. Structure.

[11] Marquenet E., Richet E. (2007). How integration of positive and negative regulatory signals by a STAND signaling protein depends on ATP hydrolysis. Mol. Cell.

[12] Marquenet E., Richet E. (2010). Conserved motifs involved in ATP hydrolysis by MalT, a signal transduction ATPase with numerous domains from *Escherichia coli*. J. Bacteriol..

[13] Reubold T.F., Wohlgemuth S., Eschenburg S. (2009). A new model for the transition of APAF-1 from inactive monomer to caspase-activating apoptosome. J. Biol. Chem..

[14] Liu P., Danot O., Richet E. (2013). A dual role for the inducer in signalling by MalT, a signal transduction ATPase with numerous domains (STAND). Mol. Microbiol..

[15] Ade J., DeYoung B.J., Golstein C., Innes R.W. (2007). Indirect activation of a plant nucleotide binding site-leucine-rich repeat protein by a bacterial protease. Proc. Natl. Acad. Sci. U.S.A..

[16] Bao Q., Lu W., Rabinowitz J.D., Shi Y. (2007). Calcium blocks formation of apoptosome by preventing nucleotide exchange in Apaf-1. Mol. Cell.

[17] Poyet J.L., Srinivasula S.M., Tnani M., Razmara M., Fernandes-Alnemri T., Alnemri E.S. (2001). Identification of Ipaf, a human caspase-1-activating protein related to Apaf-1. J. Biol. Chem..

[18] Tanabe T., Chamaillard M., Ogura Y., Zhu L., Qiu S., Masumoto J., Ghosh P., Moran A., Predergast M.M., Tromp G. (2004). Regulatory regions and critical residues of NOD2 involved in muramyl dipeptide recognition. EMBO J..

[19] Hsu J.L., Peng H.L., Chang H.Y. (2008). The ATP-binding motif in AcoK is required for regulation of acetoin catabolism in *Klebsiella pneumoniae* CG43. Biochem. Biophys. Res. Commun..

[20] Bent A.F., Mackey D. (2007). Elicitors, effectors, and R genes: the new paradigm and a lifetime supply of questions. Annu. Rev. Phytopathol..

[21] Riedl S.J., Salvesen G.S. (2007). The apoptosome: signalling platform of cell death. Nat. Rev. Mol. Cell Biol..

[22] Yuan S., Akey C.W. (2013). Apoptosome structure, assembly, and procaspase activation. Structure.

[23] Larquet E., Schreiber V., Boisset N., Richet E. (2004). Oligomeric assemblies of the *Escherichia coli* MalT transcriptional activator revealed by cryo-electron microcopy and image processing. J. Mol. Biol..

[24] Schreiber V., Richet E. (1999). Self-association of the *Escherichia coli* transcription activator MalT in the presence of maltotriose and ATP. J. Biol. Chem..

[25] Steegborn C., Danot O., Huber R., Clausen T. (2001). Crystal structure of transcription factor MalT domain III: a domain helix repeat fold implicated in regulated oligomerization. Structure.

[26] Danot O. (2010). The inducer maltotriose binds in the central cavity of the tetratricopeptide-like sensor domain of MalT, a bacterial STAND transcription factor. Mol. Microbiol..

[27] Danot O., Vidal-Ingigliardi D., Raibaud O. (1996). Two amino acid residues from the DNA-binding domain of MalT play a crucial role in transcriptional activation. J. Mol. Biol..

[28] Rasband W.S. (1997–2014). ImageJ.

[29] Sali A., Blundell T.L. (1993). Comparative protein modelling by satisfaction of spatial restraints. J. Mol. Biol..

[30] Söding J., Biegert A., Lupas A.N. (2005). The HHpred interactive server for protein homology detection and structure prediction. Nuclear Acids Res..

[31] Ebright Y.W., Chen Y., Kim Y., Ebright R.H. (1996). S-[2-(4-azidosalicylamido)ethylthio]-2-thiopyridine: radioiodinatable, cleavable, photoactivatible cross-linking agent. Bioconjugate Chem..

[32] Schlegel A., Danot O., Richet E., Ferenci T., Boos W. (2002). The N terminus of the *Escherichia coli* transcription activator MalT is the domain of interaction with MalY. J. Bacteriol..

[33] Schreiber V., Steegborn C., Clausen T., Boos W., Richet E. (2000). A new mechanism for the control of a prokaryotic transcriptional regulator: antagonistic binding of positive and negative effectors. Mol. Microbiol..

[34] Danot O. (2001). A complex signaling module governs the activity of MalT, the prototype of an emerging transactivator family. Proc. Natl. Acad. Sci. U.S.A..

[35] Richardson J.S. (1981). The anatomy and taxonomy of protein structure. Adv. Protein Chem..

[36] Dardonville B., Raibaud O. (1990). Characterization of *malT* mutants that constitutively activate the maltose regulon of *Escherichia coli*. J. Bacteriol..

[37] Notley-McRobb L., Ferenci T. (1999). The generation of multiple co-existing mal-regulatory mutations through polygenic evolution in glucose-limited populations of *Escherichia coli*. Environ. Microbiol..

[38] Hong M., Yoon S.I., Wilson I.A. (2012). Structure and functional characterization of the RNA-binding element of the NLRX1 innate immune modulator. Immunity.

[39] Reubold T.F., Hahne G., Wohlgemuth S., Eschenburg S. (2014). Crystal structure of the leucine-rich repeat domain of the NOD-like receptor NLRP1: Implications for binding of muramyl dipeptide. FEBS Lett..

[40] Tenthorey J.L., Kofoed E.M., Daugherty M.D., Malik H.S., Vance R.E. (2014). Molecular basis for specific recognition of bacterial ligands by NAIP/NLRC4 inflammasomes. Mol. Cell.

[41] Bej A., Sahoo B.R., Swain B., Basu M., Jayasankar P., Samanta M. (2014). LRRsearch: an asynchronous server-based application for the prediction of leucine-rich repeat motifs and an integrative database of NOD-like receptors. Comput. Biol. Med..

[42] Slootweg E.J., Spiridon L.N., Roosien J., Butterbach P., Pomp R., Westerhof L., Wilbers R., Bakker E., Bakker J., Petrescu A.J. (2013). Structural determinants at the interface of the ARC2 and leucine-rich repeat domains control the activation of the plant immune receptors Rx1 and Gpa2. Plant Physiol..

[43] Faustin B., Chen Y., Zhai D., Le Negrate G., Lartigue L., Satterthwait A., Reed J.C. (2009). Mechanism of Bcl-2 and Bcl-X(L) inhibition of NLRP1 inflammasome: loop domain-dependent suppression of ATP binding and oligomerization. Proc. Natl. Acad. Sci. U.S.A..

[44] Joly N., Bohm A., Boos W., Richet E. (2004). MalK, the ATP-binding cassette component of the *Escherichia coli* maltodextrin transporter, inhibits the transcriptional activator MalT by antagonizing inducer binding. J. Biol. Chem..

[45] Joly N., Danot O., Schlegel A., Boos W., Richet E. (2002). The Aes protein directly controls the activity of MalT, the central transcriptional activator of the *Escherichia coli* maltose regulon. J. Biol. Chem..

[46] Yuan S., Topf M., Reubold T.F., Eschenburg S., Akey C.W. (2013). Changes in Apaf-1 conformation that drive apoptosome assembly. Biochemistry.

[47] Malhotra V., Arteaga-Cortes L.T., Clay G., Clark-Curtiss J.E. (2010). *Mycobacterium tuberculosis* protein kinase K confers survival advantage during early infection in mice and regulates growth in culture and during persistent infection: implications for immune modulation. Microbiology.

[48] Malhotra V., Okon B.P., Clark-Curtiss J.E. (2012). *Mycobacterium tuberculosis* protein kinase K enables growth adaptation through translation control. J. Bacteriol..

[49] Jones S.A., Jorgensen M., Chowdhury F.Z., Rodgers R., Hartline J., Leatham M.P., Struve C., Krogfelt K.A., Cohen P.S., Conway T. (2008). Glycogen and maltose utilization by *Escherichia coli* O157:H7 in the mouse intestine. Infect. Immun..

[50] Chang D.E., Smalley D.J., Tucker D.L., Leatham M.P., Norris W.E., Stevenson S.J., Anderson A.B., Grissom J.E., Laux D.C., Cohen P.S. (2004). Carbon nutrition of *Escherichia coli* in the mouse intestine. Proc. Natl. Acad. Sci. U.S.A..

[51] Lang H., Jonson G., Holmgren J., Palva E.T. (1994). The maltose regulon of *Vibrio cholerae* affects production and secretion of virulence factors. Infect. Immun..

[52] Lone A.G., Deslandes V., Nash J.H., Jacques M., MacInnes J.I. (2009). *malT* knockout mutation invokes a stringent type gene-expression profile in *Actinobacillus pleuropneumoniae* in bronchoalveolar fluid. BMC Microbiol..

