# Supplementary material for: How ‘arm-twisting’ by the inducer triggers activation of the MalT transcription factor, a typical signal transduction ATPase with numerous domains (STAND)
Source: Nucleic Acids Res. 2015 Mar 3;43(6):3089–99. doi: 10.1093/nar/gkv158 (PMC4381067; doi:10.1093/nar/gkv158)
Supplement: SUPPLEMENTARY DATA [file supp_43_6_3089__index.html]

How ‘arm-twisting’ by the inducer triggers activation of the MalT transcription factor, a typical signal transduction ATPase with numerous domains (STAND) — How ‘arm-twisting’ by the inducer triggers activation of the MalT transcription factor, a typical signal transduction ATPase with numerous domains (STAND) — How ‘arm-twisting’ by the inducer triggers activation of the MalT transcription factor, a typical signal transduction ATPase with numerous domains (STAND) — SUPPLEMENTARY DATA 

# How ‘arm-twisting’ by the inducer triggers activation of the MalT transcription factor, a typical signal transduction ATPase with numerous domains (STAND)

## SUPPLEMENTARY DATA

**Files in this Data Supplement:**

- SUPPLEMENTARY DATA
