# Supplementary material for: How ‘arm-twisting’ by the inducer triggers activation of the MalT transcription factor, a typical signal transduction ATPase with numerous domains (STAND)
Source: Nucleic Acids Res. 2015 Mar 3;43(6):3089–99. doi: 10.1093/nar/gkv158 (PMC4381067; doi:10.1093/nar/gkv158)
Supplement: SUPPLEMENTARY DATA [file supp_gkv158_nar-03577-m-2014-File008.pdf]

SUPPLEMENTARY MATERIAL AND METHODS

Strains and plasmids

pOM206 (1) is a pET24a(+) (Merck-Millipore) derivative encoding an N-terminally His-tagged MalT (HMalT). pOM206*KCSI-Q70C*, pOM206*Q70C*, pOM206*E395*C, pOM206*Q70C-E395C*, respectively encoding HMalT$^{C–,Q70C}$, HMalT^{Q70C}, HMalT^{E395C} and HMalT^{Q70C,E395C}, are derived from pOM206 by classical mutagenesis and cloning techniques. Mutations introduced are described in Table S1. pOM167 is a pET24a(+) derivative encoding an N-terminally His-tagged version of the arm-sensor moiety of MalT (HMalT336-806). The sequence between the NdeI and HindIII sites of pOM167 is 5'-catatgCATCACCATCACCATCATATG-malT(1006-2418)-GGCGCCTaagctt-3' (NdeI and HindIII sites in lower case, stop codon underlined, extent of the malT coding sequence present indicated in parentheses). As a result, the encoded protein sequence is MHHHHHHM-MalT(336-806)-GA (extent of the MalT amino-acid sequence in parentheses).

Protein purification

HMalT, HMalT^{Q70C,E395C}, HMalT^{Q70C} or HMalT^{E395C} were purified as follows. Cells of pop8012 (a *malT*$^{-}$ derivative of BL21(DE3), (1)) harbouring pOM206 or derivatives thereof (see above) were grown for ~30 h in 400 ml of ZYP 5052' (i.e., ZYP5052 (2) without trace metals and with tryptone instead of N-Z amine) supplemented with 25µg/µl kanamycine in propeller flasks at 200 rpm and 20°C. Bacteria were harvested by centrifugation, washed and resuspended in 50 mM Tris-HCl pH 8.0, 10 % sucrose, 0.3 M KI (TSI) buffer. ATP was added to 0.4 mM and cells were disrupted in a French press cell at 16000 psi. Extracts were centrifuged at 160000 g for 1 h and the supernatant was loaded on a HisPrep FF 16/10 column (GE Healthcare) equilibrated in TSI + 0.4 mM ATP. The column was washed with TSI + 0.4 mM ATP (7 CV) , then with TSI + 30 mM imidazole, 0.4 mM ATP (3 CV) and eluted with TSI + 200 mM imidazole + 0.4 mM ATP. The protein was further purified on a Superdex 200 XK26/60 (GE Healthcare) equilibrated in buffer A (50 mM Tris-HCl pH 8.0, 10 % sucrose, 0.2 M KI, 10 mM Mg acetate, 0.1 mM EDTA) + 200 µM $Na_2S_2O_3$ + 0.4 mM ATP. The protein was concentrated to 10-20 mg/ml and flash-frozen.

HMalT$^{C–,Q70C}$ was purified in the same way, except for the following modifications. Cultures were grown for ~45h at 16°C, 0.5 mM ATP was used instead of 0.4 mM, the columns were a 5 mL HisTrap and a 10/300GL superdex 200 (both from GE Healthcare) and proteins were concentrated to 3.8-4.4 mg/mL. Buffer was changed at the HisTrap imidazole wash step to B (50 mM Tris-HCl (pH 8.0), 10 % sucrose, 0.3 M KCl, 10 mM Mg acetate, 0.1 mM EDTA) + 30 mM imidazole + 0.5 mM ATP. HisTrap elution was carried out in B + 200 mM imidazole + 0.5 mM ATP, and the superdex 200 column was run in B + 0.5 mM ATP.

For HMalT$^{336\text{-}806}$, BL21(DE3) cells harbouring pOM167 were grown in 2 L LB medium supplemented with 0.4% maltose and 25 µg/µl kanamycine at 37°C. At OD^{600} = 2.5, isopropyl-thio-ß-D-galactoside was added to 0.1 mM and cultures were further grown for 4 h at 30 °C. Cells were harvested by centrifugation and resuspended in TSI buffer. Maltotriose was added to 2 mM and cells were disrupted in a French press cell at 16000 psi. Extracts were centrifuged at 260000 g for 1 h and

the supernatant was loaded onto a HisPrep FF 16/10 column (GE Healthcare) equilibrated in TSI + 1 mM maltotriose. The column was washed with TSI + 1 mM maltotriose (7 CV) , then with 50 mM Tris-HCl (pH 8.0), 10 % sucrose, 0.5 M KCl containing 30 mM imidazole + 1mM maltotriose (5 CV) and eluted with the same buffer containing 200 mM imidazole + 1mM maltotriose. Half of the pooled Hisprep fractions were further purified on a Superdex 200 XK26/60 (GE Healthcare) equilibrated in 50 mM Tris-HCl (pH 8.0), 10 % sucrose, 0.3 M KCl, 1 mM maltotriose. Fifteen mg of the eluate were filtered through a HiTrap Desalting column (GE Healthcare) equilibrated in a 20 mM Tris-HCl (pH 8.0), 10 % sucrose, 0.2M KCl buffer, concentrated to 300 µL and dialyzed overnight against the same buffer (2 x 500mL, one buffer change).

SDS-PAGE analysis of the disulfide bond

Proteins (30 µL, 11.7 mg/mL) in buffer A + 200 µM $Na_2S_2O_3$ + 0.4 mM ATP were denatured by passing successively through two 0.7 mL Protein Desalting Spin Columns (Thermo Fisher Scientific, USA) equilibrated with 50 mM sodium phosphate buffer (pH 7.0) containing 8M guanidinium hydrochloride, and divided in two. One part was left untreated, and the other was treated with 5 mM N-ethylmaleimide (NEM) for 30 min at 20°C. NEM-treated and non-treated samples were precipitated with 32.3 volumes of 16.5% TCA. The pellet was washed twice with cold acetone and dried. In an additional experiment, the protein was pretreated with 2 mM DTT for 15 min at room temperature as a first step, and handled exactly as the other samples after that. Samples were redissolved in sample buffer ± 70 mM DTT, boiled for 2.5 min and 1.4 µg were loaded on a 10% polyacrylamide gel. Samples with DTT were separated from samples without DTT by two empty lanes to avoid DTT contamination by diffusion during electrophoresis. Croppings of the photograph of a unique gel corresponding to +DTT and -DTT samples are shown side by side in Fig. 1E.

Endoproteinase LysC proteolysis

The protein (0.8 mg/ml) was incubated with endoproteinase LysC (Roche Applied Sciences), at a protease/protein weight ratio 1:8, in 50 mM 4-(2-hydroxyethyl-1-piperazineethanesulfonic acid) (HEPES, pH 8.0), 50 mM Tris-HCl (pH 8.5), 20 mM Tris-HCl (pH 8.0), 10 mM EDTA, 0.1% sodium dodecyl sulfate, 4% sucrose, 120 mM KCl, 4 mM Mg acetate, 0.4 mM maltotriose, 0.2 mM ATP, for 72-96 h at 37°C. Samples were diluted in sample buffer with or without 70 mM DTT, boiled for 2.5 min and analysed by SDS-PAGE on Tricine gels (3).

Analysis of the cross-linking of HMalT$^{C-,Q70C}$ by AET

Fifteen µg of AET cross-linked protein that had been digested by endoproteinase LysC were analyzed on a 16% SDS-PAGE Tricine gel followed by transfer on a PVDF membrane. The 18 kDa band characteristic of the non-reduced pattern was excised and subjected to N-terminal microsequencing in a Procise Model 494 apparatus (Applied Biosystems). The result was a mix of two sequences. Comparison with the theoretical fragments expected from the specificity of endoproteinase

LysC unambiguously identified the sequences as TTLI and SAIL. The 18 kDa band thus contained a mix of fragments originating at positions 46 and 274 of the wt sequence. The simplest interpretation of these results is that endoproteinase LysC digestion was incomplete and that the 18 kDa band contained the 46-99 fragment (5924 Da, containing C70) cross-linked to the 274-398 fragment (14364 Da). This was consistent with the fact that a 14 kDa fragment was depleted in the non reduced lane (Fig.1D).

To narrow down the location of the cross-link(s), the 18 kDa and the 14 kDa fragments stemming from the endoproteinase LysC digestion of 63 µg AET-cross-linked HMalT$^{C-,Q70C}$ protein were excised from a Tricine gel run in non-reducing conditions and digested overnight at 30°C by 2 ng/µl sequencing-grade trypsin (Promega) in a buffer containing 50 mM Tris-HCl (pH 8.6) and 0.01% Tween 20. Peptides were separated by HPLC on DEAE-C18 inline columns (1 mm diameter) with a 2%-70% acetonitrile gradient in 0.1% trifluoroacetic acid.

HPLC profiles of the 14 kDa and 18 kDa species were similar, except for one major peak, 18C, which was only present in the 18 kDa profile. Peak 18C and the 5 major peaks (14A to E) of the 14kDa profile, as a control, were analyzed by SELDI-TOF. Two µL of these peaks were spotted on Gold Chips (Ciphergen, USA) with an α-cyano-4-hydroxycinnamic acid matrix and analyzed in a Protein Chipreader System 4000 (Ciphergen), with a focus mass of 4000 Da. 18C gave two major mass peaks in the 4500-6000 Da range (Fig. S1B). These masses corresponded to those expected for an AET cross-link between the 57-73 trypsin fragment and either the 346-374 peptide of HMalT$^{C-,Q70C}$ or the 375-398 peptide with one methionine oxidized (Fig. S1C, using average masses and assuming that the cross-linking reaction occurs as indicated by Ebright et *al.* (4), which gives an additional mass of 208.2 for the cross-linking arm). Microsequencing of the 18C peak confirmed the presence of the N-termini of these 3 peptides (Fig. S1D). No significant mass peak was observed in the 4500-6000 Da range in any spectrum from the 14 kDa peptide (Fig. S1B).

SUPPLEMENTARY TABLE

mutation	nucleotide and amino acid sequence[1]	residue substitution	silent restriction site[2]
KCSI	89 H S A I S E<br>265 CACTCCGCGATATCCGAG	C90S<br>C93S	EcoRV
	196 D S R L S S P I E A A E S S R I S D<br>586 GATTCCCGTCTGAGCTCGCCGATTGAAGCCGCAGAAAGCAGTCGGATTTCCGAT	C197S<br>C212S	SacI
	318 F S Y H P L F G N F L R Q R S Q<br>952 TTCTCCTATCACCCGCTGTTTGGTAACTTCCTGCGCCAGCGCAGCCAG	C319S<br>C332S	
	499 H S K G<br>1495 CACTCCAAGGGC	C500S	StyI
	610 Q S L A M L I Q I S<br>1828 CAGTCCCTGGCAATGTTGATTCAGATCTCG	C611S<br>C618I	BglII
	825 P E V P E<br>2473 CCTGAGGTACCTGAA		KpnI
Q70C	69 N C Q<br>205 AACTGCCAA	Q70C	
E395C	393 L E C S<br>1177 CTCGAGTGCTCG	E395C	XhoI

Table S1 Sequence changes in the *malT* sequence generated by mutations *KCSI*, *Q70C* and *E395C*

[1]Nucleotide sequences encompassing the mutations are given, with the changes underlined, and the corresponding amino acid sequence above. Nucleotide and amino acid sequence are numbered after the sequence of the wt *malT* gene and of the wt MalT protein, respectively

[2]Silent restriction sites inserted for technical reasons are indicated.

SUPPLEMENTARY REFERENCES

1. Marquenet, E. and Richet, E. (2007) How integration of positive and negative regulatory signals by a STAND signaling protein depends on ATP hydrolysis. Mol. Cell, 28, 187-199.
2. Studier, F.W. (2005) Protein production by auto-induction in high density shaking cultures. Protein expression and purification, 41, 207-234.
3. Schägger, H. (2006) Tricine-SDS-PAGE. Nature protocols, 1, 16-22.
4. Ebright, Y.W., Chen, Y., Kim, Y. and Ebright, R.H. (1996) S-[2-(4-azidosalicylamido)ethylthio]-2-thiopyridine: radioiodinatable, cleavable, photoactivatible cross-linking agent. Bioconjugate chemistry, 7, 380-384.

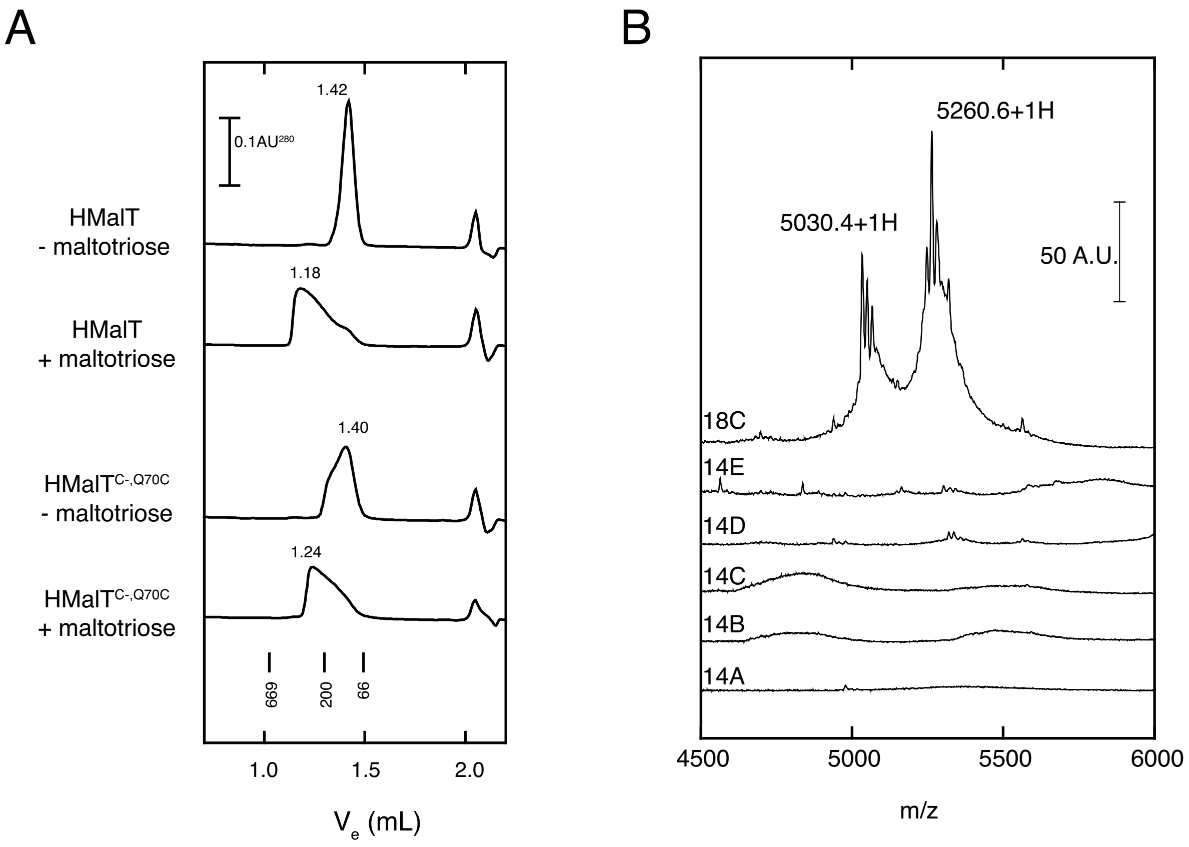

C

peptide	expected mass (for AET cross-link with 57-73, in Da)
375-398	5031.5
346-374^{ox}	5261.7

D

^{57}NDIGWY

^{346}AAAESW

^{375}DILLNHA

Fig. S1 Characterization of the AET cross-links of HMalT$^{C-,Q70C}$. A. Inducer-dependent multimerization of the HMalT and HMalT$^{C-,Q70C}$ proteins. Proteins (15 μM) were run through a Superdex 200 exclusion chromatography column in a buffer containing 50 mM Tris-HCl (pH 7.7), 0.3 M KCl, 10 mM Mg acetate, 0.1 mM EDTA and 0.5 mM ATP, with or without 1 mM maltotriose after 15 min preincubation in the column buffer supplemented with 10 % sucrose. B. Mass spectra of the 18 C peak and of the major peaks from the 14 kDa HPLC profile (A. U., arbitrary units). C. Predicted masses for AET cross-links of the 57-73 peptide with indicated peptides of the arm. ^{ox} stands for oxidated (+16 Da). D. N-terminal sequences of the fragments contained in the 18C HPLC peak.

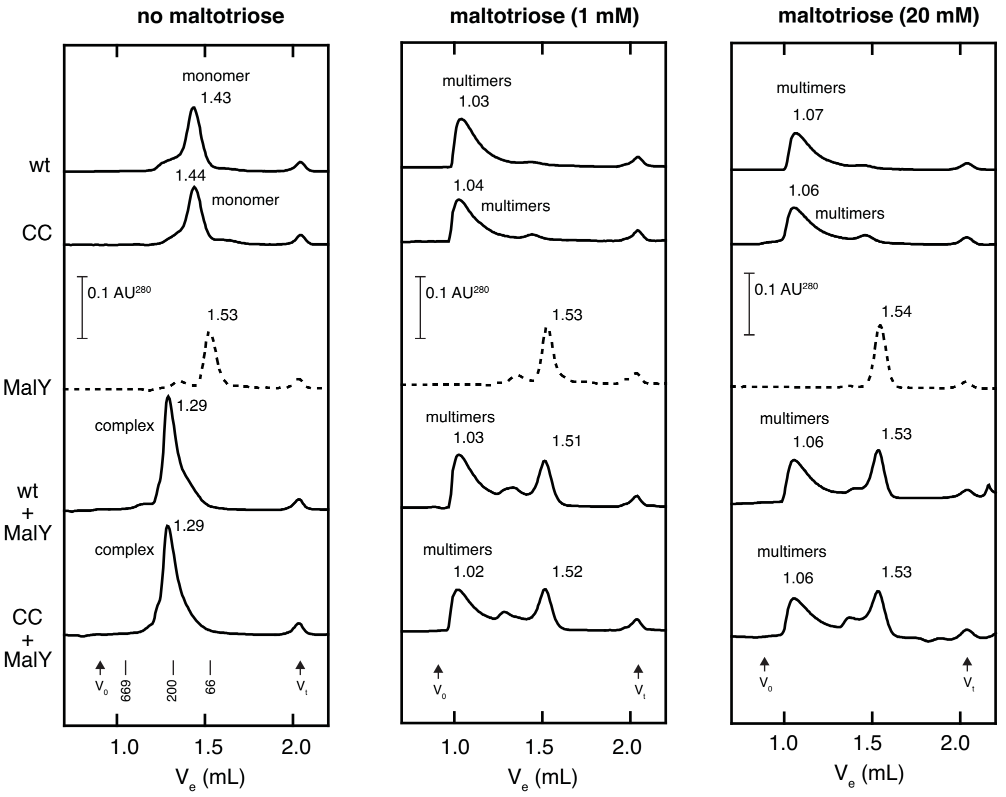

Fig. S2. The properties of oxidized HMalT^{Q70C,E395C} in the presence of inducer (monomeric and able to form a complex with MalY) are not a consequence of the Q70C and E395C substitutions per se. Reduced HMalT^{Q70C,E395C}, like HMalT, multimerizes in response to maltotriose and forms a complex with MalY that dissociates upon maltotriose addition. Conditions were as in Fig.3, except that 3 mM and 1 mM DTT were added in the preincubation and filtration buffers, respectively.

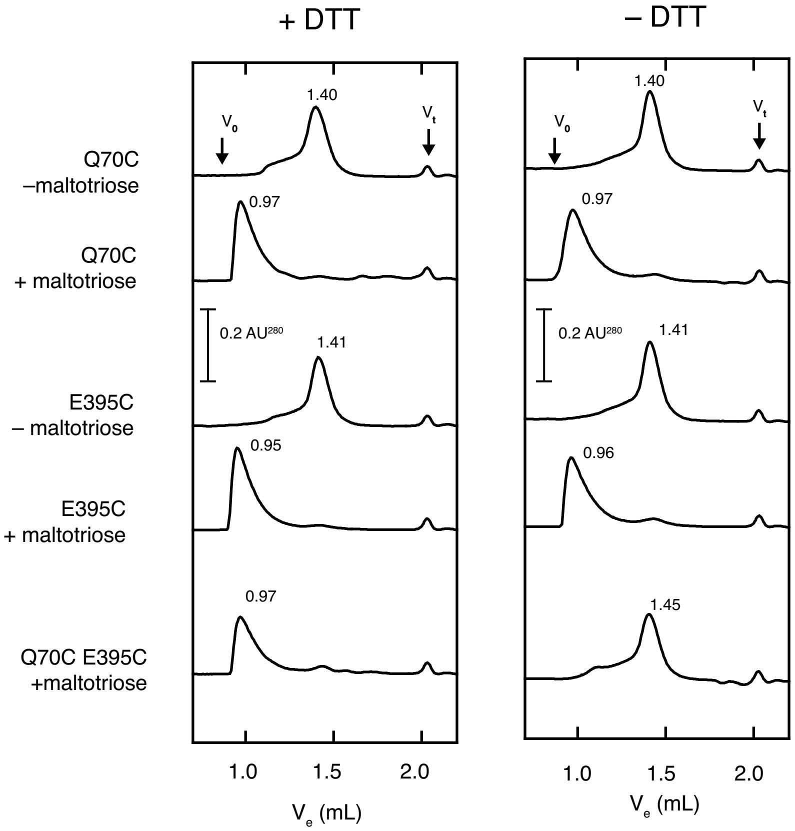

Fig. S3 The absence of inducer-dependent multimerization observed for oxidized $HMalT^{Q70C,E395C}$ requires both Q70C and E395C substitutions. Like HMalT, non-reduced $HMalT^{Q70C}$ and $HMalT^{E395C}$ multimerize in response to maltotriose, by contrast to non-reduced $HMalT^{Q70C,E395C}$. Conditions were as in Fig. 3, except that protein concentrations were 25 μM, which explains the difference in elution volume of the multimers compared to Fig. 3 and S2, due to the formation of higher order assemblies. Maltotriose (1 mM in the running and incubation buffers) and DTT (1 mM in the running buffer and 3 mM in the incubation buffer) were present when indicated.

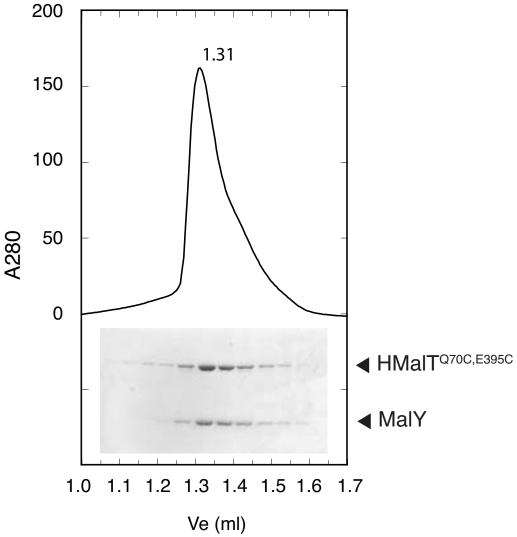

Fig. S4 The peak obtained by cofiltration of HMalT^{Q70C,E395C} and MalY in non-reducing conditions in the presence of 20 mM maltotriose (Fig. 3 bottom right curve) contains an HMalT^{Q70C,E395C}-MalY complex. Fractions (50μL) were collected during the HMalT$^{Q70C-E395C}$+MalY (– DTT + 20 mM maltotriose) filtration of Fig. 3. Five μl were analyzed by SDS-PAGE after Coomassie Blue staining.

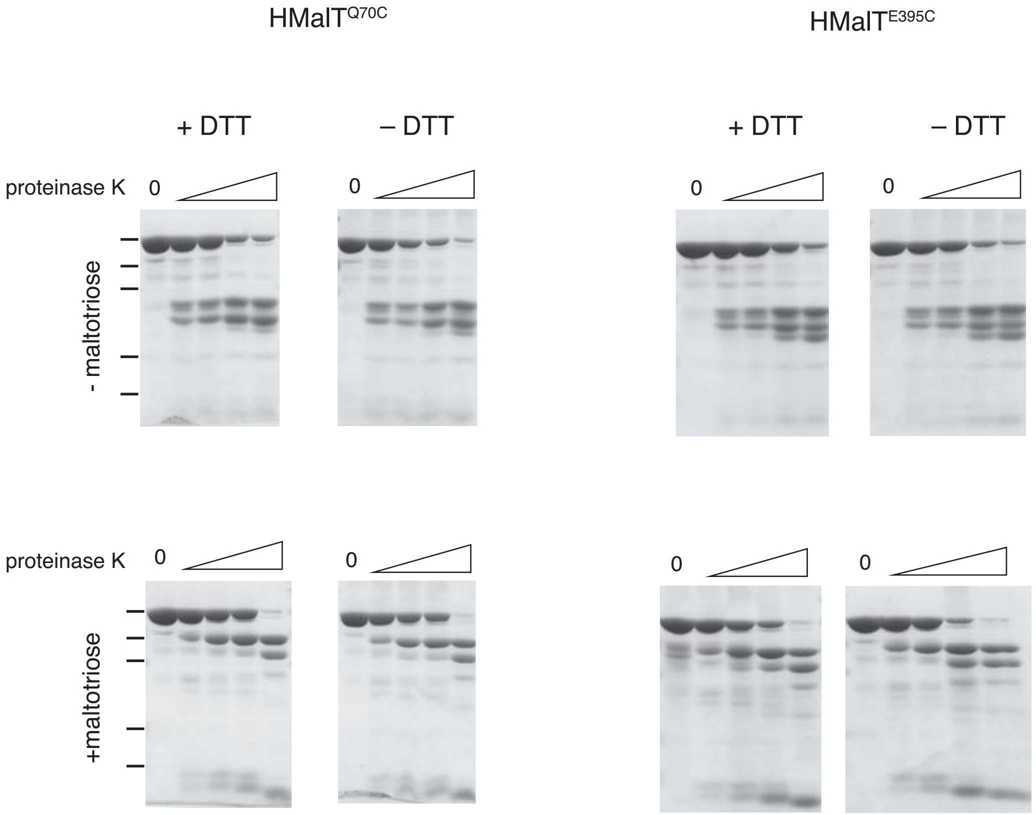

Fig. S5 Limited proteolysis of HMalT^{Q70C} and HMalT^{E395C} by proteinase K with or without inducer under reducing or non-reducing conditions. Proteinase K:HMalT (w/w) ratios were as in Fig. 4A. Black dashes indicate the position of protein markers of 100, 70, 55, 35 and 25 kDa.

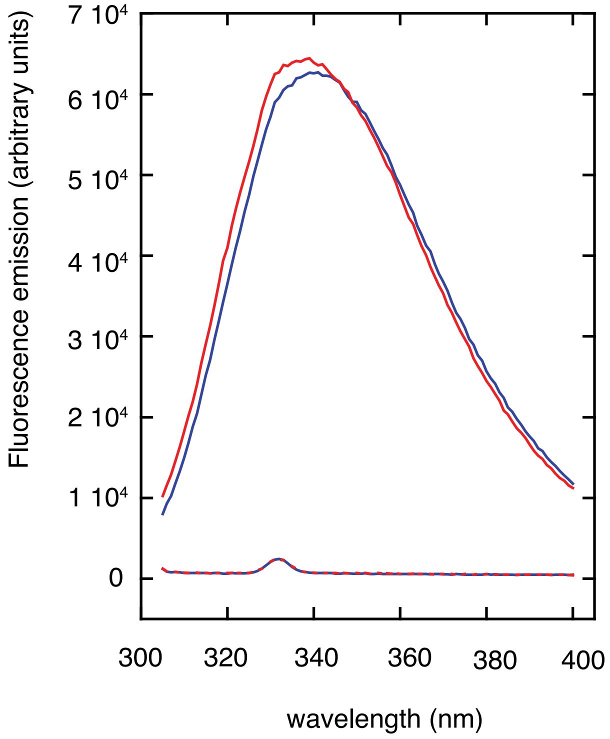

Fig. S6 Intrinsic fluorescence spectrum of HMalT (1μM) in 20 mMTris-HCl (pH 7.9) at 20°C in the presence (red curve) and absence (blue) of 60 μM maltotriose. Excitation wavelength was 295 nm. The dotted lines represent the spectrum of the buffer, with (red) or without (blue) 60 μM maltotriose
